# Multiple genome pattern analysis and signature gene identification for the Caucasian lung adenocarcinoma patients with different tobacco exposure patterns

**DOI:** 10.7717/peerj.8349

**Published:** 2020-01-30

**Authors:** Yan-mei Dong, Li-da Qin, Yi-fan Tong, Qi-en He, Ling Wang, Kai Song

**Affiliations:** 1 School of Chemical Engineering and Technology, Tianjin University, Tianjin, China; 2 The First Affiliated Hospital Oncology, Dalian Medical University, Dalian, Liaoning, China

**Keywords:** Multiple genome, Lung adenocarcinoma, Precision Medicine, Tobacco-related signature genes

## Abstract

**Background:**

When considering therapies for lung adenocarcinoma (LUAD) patients, the carcinogenic mechanisms of smokers are believed to differ from those who have never smoked. The rising trend in the proportion of nonsmokers in LUAD urgently requires the understanding of such differences at a molecular level for the development of precision medicine.

**Methods:**

Three independent LUAD tumor sample sets—TCGA, SPORE and EDRN—were used. Genome patterns of expression (GE), copy number variation (CNV) and methylation (ME) were reviewed to discover the differences between them for both smokers and nonsmokers. Tobacco-related signature genes distinguishing these two groups of LUAD were identified using the GE, ME and CNV values of the whole genome. To do this, a novel iterative multi-step selection method based on the partial least squares (PLS) algorithm was proposed to overcome the high variable dimension and high noise inherent in the data. This method can thoroughly evaluate the importance of genes according to their statistical differences, biological functions and contributions to the tobacco exposure classification model. The kernel partial least squares (KPLS) method was used to further optimize the accuracies of the classification models.

**Results:**

Forty-three, forty-eight and seventy-five genes were identified as GE, ME and CNV signatures, respectively, to distinguish smokers from nonsmokers. Using only the gene expression values of these 43 GE signature genes, ME values of the 48 ME signature genes or copy numbers of the 75 CNV signature genes, the accuracies of TCGA training and SPORE/EDRN independent validation datasets all exceed 76%. More importantly, the focal amplicon in Telomerase Reverse Transcriptase in nonsmokers, the broad deletion in ChrY in male nonsmokers and the greater amplification of MDM2 in female nonsmokers may explain why nonsmokers of both genders tend to suffer LUAD. These pattern analysis results may have clear biological interpretation in the molecular mechanism of tumorigenesis. Meanwhile, the identified signature genes may serve as potential drug targets for the precision medicine of LUAD.

## Introduction

Lung cancer is currently the most common malignant disease and the leading cause of mortality in the world (Siegel, Miller & Jemal, 2018; Tian, 2019). Lung adenocarcinoma (LUAD) and lung squamous cell carcinoma are the two major histological types of non-small cell lung cancer (NSCLC) covering over 90% of cases; NSCLC itself accounts for 75–80% of lung cancer cases (Qiu et al., 2017; Chen et al., 2015; Tian, 2015). Large cell cancer samples represent undifferentiated NSCLCs that do not show morphological or immunostaining evidence of glandular or squamous differentiation.

Smokers are 15–30 times more likely to get lung cancer or die from lung cancer than nonsmokers. Even though tobacco smoking is the major risk for lung cancer, there are still 10–15% of cancer patients of western world who have no history of tobacco exposure (Jemal et al., 2011; Lam et al., 2007). Importantly, the proportion of nonsmokers has been rising. Nonsmoker lung cancer patients consist mostly of women who tend to suffer LUAD (Liu et al., 2006; Choi et al., 2019). Environmental and occupational exposures, as well as genetic susceptibility, are thought to contribute to lung cancer risk in never-smokers (Imielinski et al., 2012; Govindan et al., 2012; Alexandrov et al., 2013).

There is increasing evidence for many different patterns in gene expressions (GE), copy number variations (CNV) and methylation (ME) values between smokers and nonsmokers (Powell et al., 2003; West et al., 2004; Li et al., 2010; Karlsson et al., 2014). Therefore, efforts to reveal the differences in tumor initiation mechanisms between smokers and nonsmokers, progression and prognosis in LUAD have been one of the hottest topics of precision medicine research for many years (Jang et al., 2012; Subramanian & Govindan, 2007; Kim et al., 2013).

In our work, therefore, the aim is two-fold: (1) an overview comparison of genome-wide GE, CNV and ME pattern analysis between current smokers and nonsmokers to discover the important differences between them; and (2) to explore tobacco-related GE, CNV and ME signature gene identifications to accurately distinguish smokers from nonsmokers.

We shall use the following definitions for tobacco usage and exposure (Song et al., 2018):

- Current smoker. An adult who has smoked at least 100 cigarettes in their lifetime and who currently smokes cigarettes or has quit within the previous 12 months.
- Former (“reformed”) smoker. An adult who has smoked at least 100 cigarettes in their lifetime but has quit smoking for longer than the previous 12 months.
- Never smoker. An adult who has never smoked, or has smoked less than 100 cigarettes in their lifetime.
- Ever smoker. An adult who has smoked at least 100 cigarettes in their lifetime (irrespective of whether they are currently smoking).

## Materials and Methods

### Datasets

Three independent LUAD datasets were used in our study (summarized in Table 1) as follows:

#### TCGA data

The Cancer Genome Atlas (TCGA) project (Legacy GDC) data is a public dataset hosted by TCGA (https://portal.gdc.cancer.gov/projects, Access Dates: 06/2017–08/2017). The level three GE, CNV and ME data of LUAD patients were used in our study. They were collected via the Illumina HiSeq 2000 RNA Sequencing Version 2, Affymetrix Genome-Wide Human Single Nucleotide Polymorphisms (SNP) Array 6.0 and Human Methylation 450 platform, respectively. Among these patients, only 5.8% of them are non-Caucasian.

#### EDRN data

The EDRN GE and ME datasets used as validation data were downloaded from the EDRN Public Portal (http://edrn.nci.nih.gov/, Access Dates: 06/2017–08/2017) (Selamat et al., 2012). They are publicly available and can be obtained from the NCBI Gene Expression Omnibus (GEO) (http://www.ncbi.nlm.nih.gov/geo/) with the accession number GSE32867 (GSE32867–GPL6884 for expression and GSE32867–GPL8490 for ME). GE data was collected via the Illumina Human WG-6 v3.0 expression Beadchip. ME data was collected via the Illumina HumanMethylation27_270596_v.1.2 BeadChip. Because there is no “current smoker” sample category in EDRN GE data, the “ever smokers” category was used instead. Less than one quarter of EDRN patients are Asian, others are Caucasian.

#### SPORE dataset

The SPORE CNV data used as the independent validation dataset was downloaded from GEO database (GSE74948, access dates: 06/2017–08/2017). It was measured by the Agilent 244 K Chip by Myriad Genetics (Salt Lake City, UT, USA) (Tang et al., 2013). For the SPORE dataset, only “ever and never” smoking history are provided, therefore, the patients were divided into “ever smoker” or “nonsmoker” groups. Less than 11.5% of the patients whose race information is available are non-Caucasian.

**Table 1:** Summary of samples in all datasets.

	TCGA*			EDRN		SPORE	
	GE	CNV	ME	GE	ME	GE	CNV
Sample number	359	378	323	69	102	143	106
Smoking history							
Never	59	61	54	30	30	23	14
Current	82	87	73		38		
Ever	218	230	196	39	34	120	92

**Note:**

* For TCGA, Ever smokers are combined by Former smokers >=15 years and Former smokers <15 years. For SPORE dataset, Ever smokers include current smokers.

### Preprocessing of the genome-wide sequencing and microarray data

For all datasets genes without known symbols or that could not be matched with the official Human Genome Organization symbols were removed. Genes whose GE, CNV or ME values were missing in all samples were removed (the missing ratios in all datasets were less than 5%). To make best use of the available information, genes whose values were missing in only part of the samples were still used in our study. Genes that were common to both training and validation datasets were used for signature identification analysis. The GE data was log2 transformed and the CNV data was log2 (CNV/2) transformed. Additionally, patient samples in TCGA training data who were lacking any of the important clinical parameters (age, gender, cancer stage and vital status) were removed (less than 10%).

Recent research supports the belief that DNA MEs in the promoter regions of genes have a bigger influence on gene functions than those in other regions (Jones & Laird, 1999; Jones, 1999). Therefore, for the TCGA and EDRN ME data, we used the average ME value of the SNPs located in a gene’s promoter area as its ME value.

As a result, 17,493 genes in the GE data, 23,494 genes in the CNV data and 13,564 genes in the ME data were available for the study.

### Statistical analysis

For brevity, we introduced the symbol “→” to indicate prediction scenarios. For example, “GE→tobacco” stands for predicting tobacco exposure patterns using the mRNA expression values of genes; “CNV→tobacco” is for tobacco exposure prediction using CNVs of genes and “ME→tobacco” stands for using the ME values of genes.

#### Significance analysis of microarrays algorithm

Significance analysis of microarrays (SAM) is a useful method for microarray analysis. It identifies genes with statistically significant changes in expression by assimilating a set of gene-specific *t*-tests. In SAM, the False Discovery Rate (FDR) is explored to control the family-wise error of the significance test. It is presented as a *q* value for each gene in the final list of significant genes (Tusher, Tibshirani & Chu, 2001; Larsson, Wahlestedt & Timmons, 2005).

We used a cutoff value of FDR of 0.1, as is standard in other studies.

#### Partial least squares

Partial least squares (PLS) is a widely used algorithm for modeling relationships between sets of observed variables by means of latent variables. It comprises regression and classification tasks as well as dimension reduction and modeling (Rosipal & Krämer, 2006; Tan et al., 2004). Instead of finding hyperplanes of minimum variance between the response and independent variables, it finds a linear regression model by projecting the predicted variables (i.e., classification labels) and the observed variables (GE, CNV or ME values of genes in our case) to a new lower space (Nagata, Bueno & Poppi, 2002; Le Bihan, Pavo & Marchand, 2014; Wang et al., 2006). Therefore, it performs very well for the analysis of high-dimension-small-sample data in bioinformatics.

#### Kernel partial least squares

The Kernel PLS (KPLS) method is an effective generalized algorithm of PLS to improve the classification accuracy by the introduction of kernel transformation (Rosipal & Krämer, 2006). It can efficiently deal with the nonlinearity using nonlinear kernel functions (Tang et al., 2010; Kim, Lee & Lee, 2005). It is a very effective regression approach since the optimization of its parameters are simpler than other methods (Bennett & Embrechts, 2003). In this work, GE, CNV or ME values of identified signature genes were used as input variables to the nonlinear KPLS models to improve classification accuracy.

### Signature gene identification by optimizing tobacco exposure classification performance

**Figure 1:**
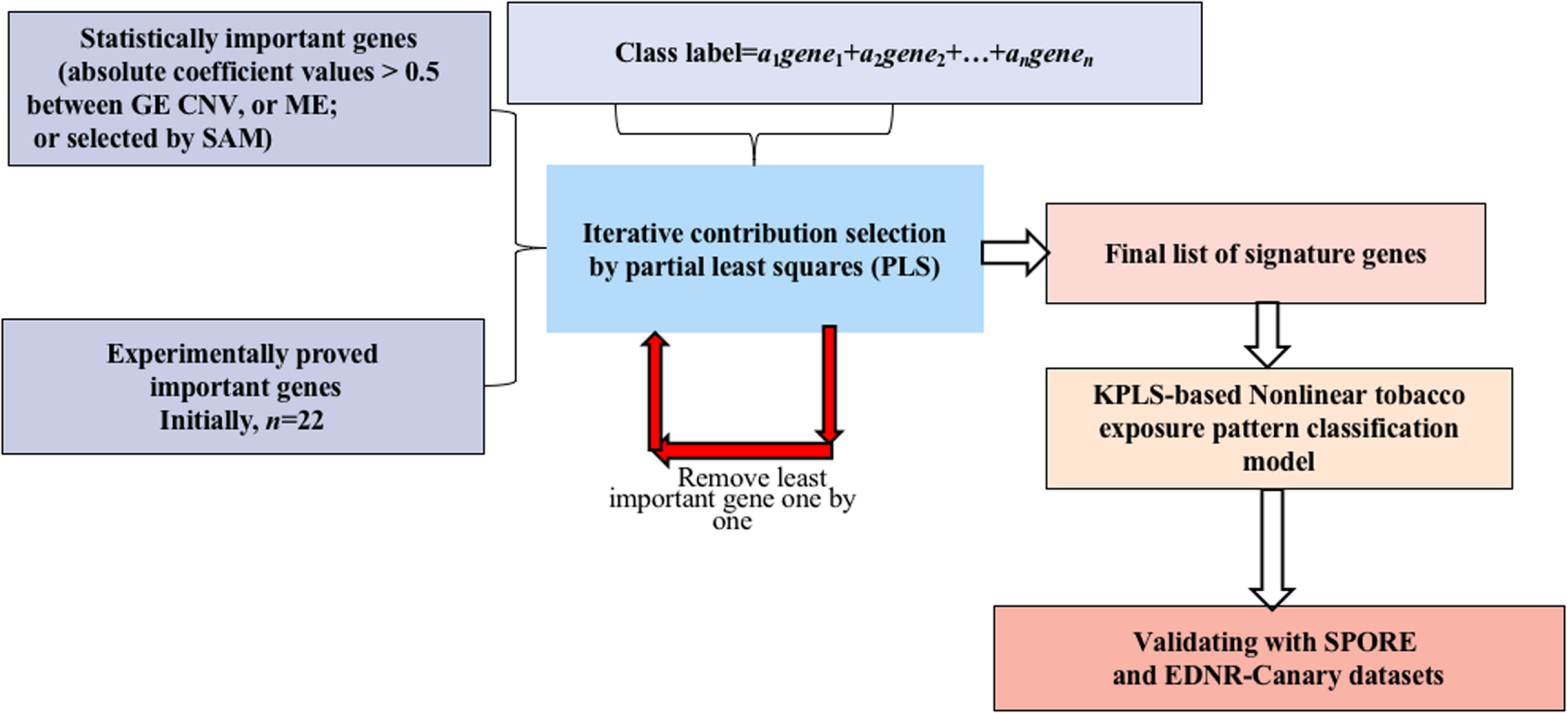
Flowchart of the integrated identification method for signature genes.

A novel method integrating statistical selection, experimental selection and iteratively contribution selection methods together is proposed. The method selects potential signature genes out of approximately 20,000 genes to distinguish smokers from nonsmokers and to overcome the disadvantages caused by high gene dimension and high noise inherent in microarray or sequencing data. This integrated identification algorithm is described below with the corresponding flowchart (Fig. 1).

#### Statistical selection of important genes

For GE data, the genes with statistically significant changes between smokers/nonsmokers in expression patterns were selected by the SAM method (FDR > 0.1) (Larsson, Wahlestedt & Timmons, 2005; Zhang et al., 2014).

For CNV and ME data, when variations in DNA MEs or copy numbers were highly correlated to their gene expression values, it is less likely for them to be caused by random noise (Gan et al., 2013; Stranger et al., 2007; Bussey et al., 2006). Therefore, genes whose MEs or copy numbers had strong relationships with GE (absolute correlation coefficient values >0.5) were selected for candidate genes.

#### Experimental selection of important genes

Several genes have been shown experimentally to be highly related to LUAD (see published papers in Table S1). These genes may play important roles in tobacco exposure classification. Selected important genes were combined with the candidate signature genes selected in the above step for further identification refinement, and to avoid the genes being overwhelmed by more than 20,000 genes.

#### Iterative contribution selection of important genes

To identify signature genes from the different scenarios, viz., GE→tobacco, CNV→tobacco and ME→tobacco, models were built first. In each model, the absolute value of the coefficient of each variable is a reasonable measurement for its contribution (Song, 2012). For each gene, the larger its contribution, the more important it is to the prediction of the tobacco exposure pattern. To overcome the overwhelming influence of gene dimension over sample dimension, the least important genes were removed one by one and the contributions of the remaining genes were reevaluated by remodeling the classification model. These two steps were repeated iteratively until the tobacco exposure pattern classification accuracy could not be further improved. Then, the remaining genes were considered as the signature genes since by using only their GE, CNV or ME values we could achieve the highest accuracy for classifying the tobacco exposure patterns of the LUAD samples.

Partial least squares was used as the modeling algorithm. Five fold cross-validation was explored to train and optimize the classification model. Accuracy, sensitivity and specificity were used to assess classification performance. The flowchart of smoking pattern signature gene identification and the classification model optimization is shown in Fig. 1. All analyses were performed using R (version 3.2.3 2015-12-10, https://www.r-project.org/alt-home/) codes. Further details of all methods and algorithms are provided in the Supplemental Materials.

### Kyoto Encyclopedia of Genes and Genomes

Kyoto Encyclopedia of Genes and Genomes (KEGG) is a knowledge base for systematic analysis of gene functions at the molecular-level in biological systems, from cells to organisms and ecosystems. It has been generated by genome sequencing and other high-throughput experimental technologies (Kanehisa & Goto, 2000). The online bioinformatics resource, DAVID v6.7 (Available at http://david.abcc.ncifcrf.gov/) was used to perform the KEGG pathway analysis for all signature genes.

## Results

**Figure 2:**
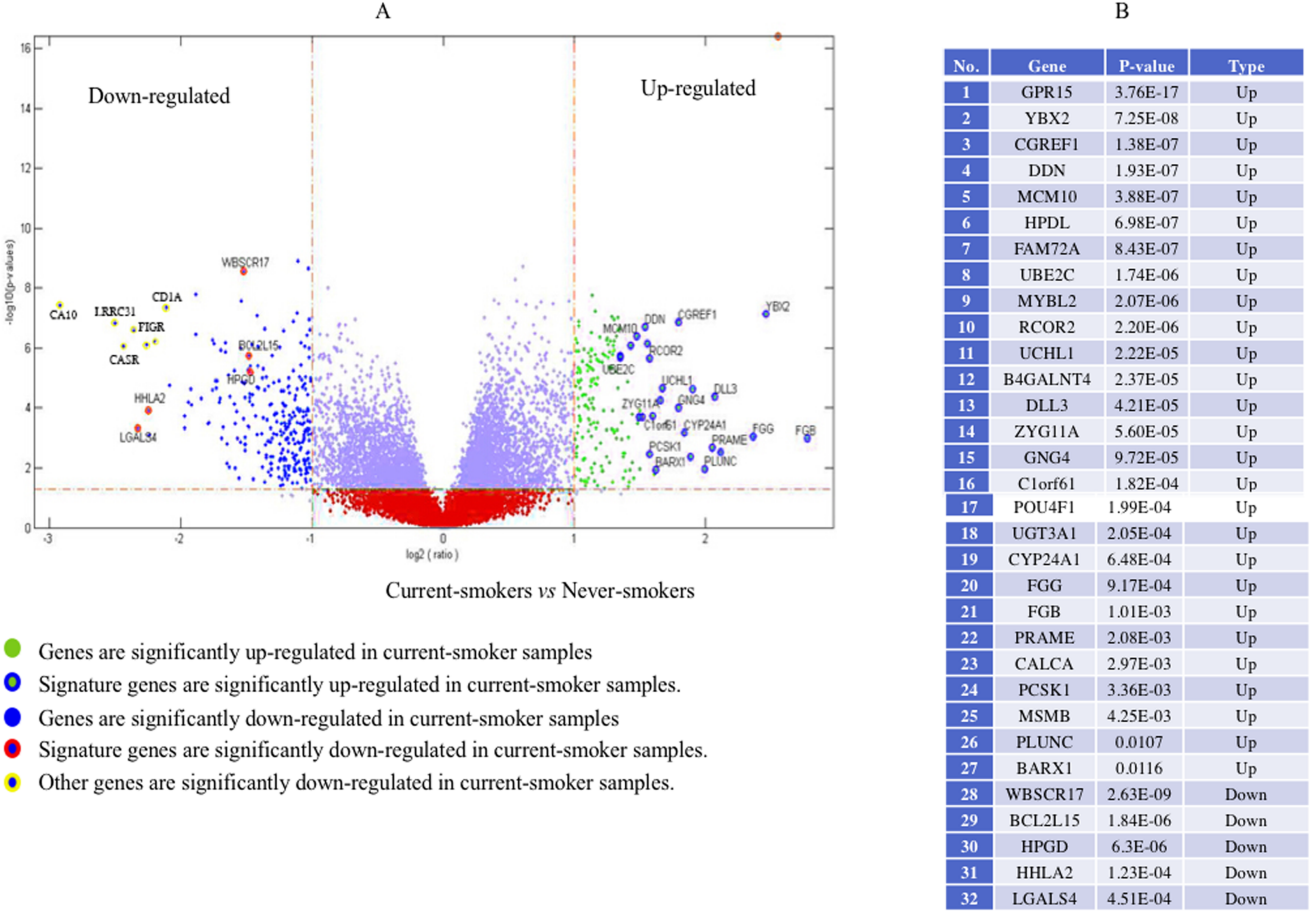
Volcano plot of genome-wide gene expression values in the TCGA dataset. (A) The *x*-axis is the log of the fold change between the expression values in current smokers vs. never-smokers. The *y*-axis is the negative log of the *p*-value on base 10 on these two groups. The cutoff of *p*-value was 2.34E−6 (Bonferroni-correction for multiple comparisons, for 21,342 genes) −log10 (2.34E−6) = 5.63. (B) The list of thirty-two out of forty-three identified GE signature genes which are significantly different with large fold changes in gene expression values between these two groups.

Genome-wide gene expression patterns, shown in the volcano plot in Fig. 2, differed greatly between current smokers vs. nonsmokers in the TCGA LUAD samples even after using a Bonferroni-correction, which is the most conservative family-wise control (Qiu et al., 2017). Thirty-two out of forty-three identified GE signature genes (Table S2) are significantly different with large fold changes in gene expression values between the two groups. Among them, GPR15, FGG and another 25 genes are significantly up-regulated in current smokers while only WBSCR17, BCL2L15, HPGD, HHLA2 and LGALS4 are down-regulated in current-smoker samples.

**Figure 3:**
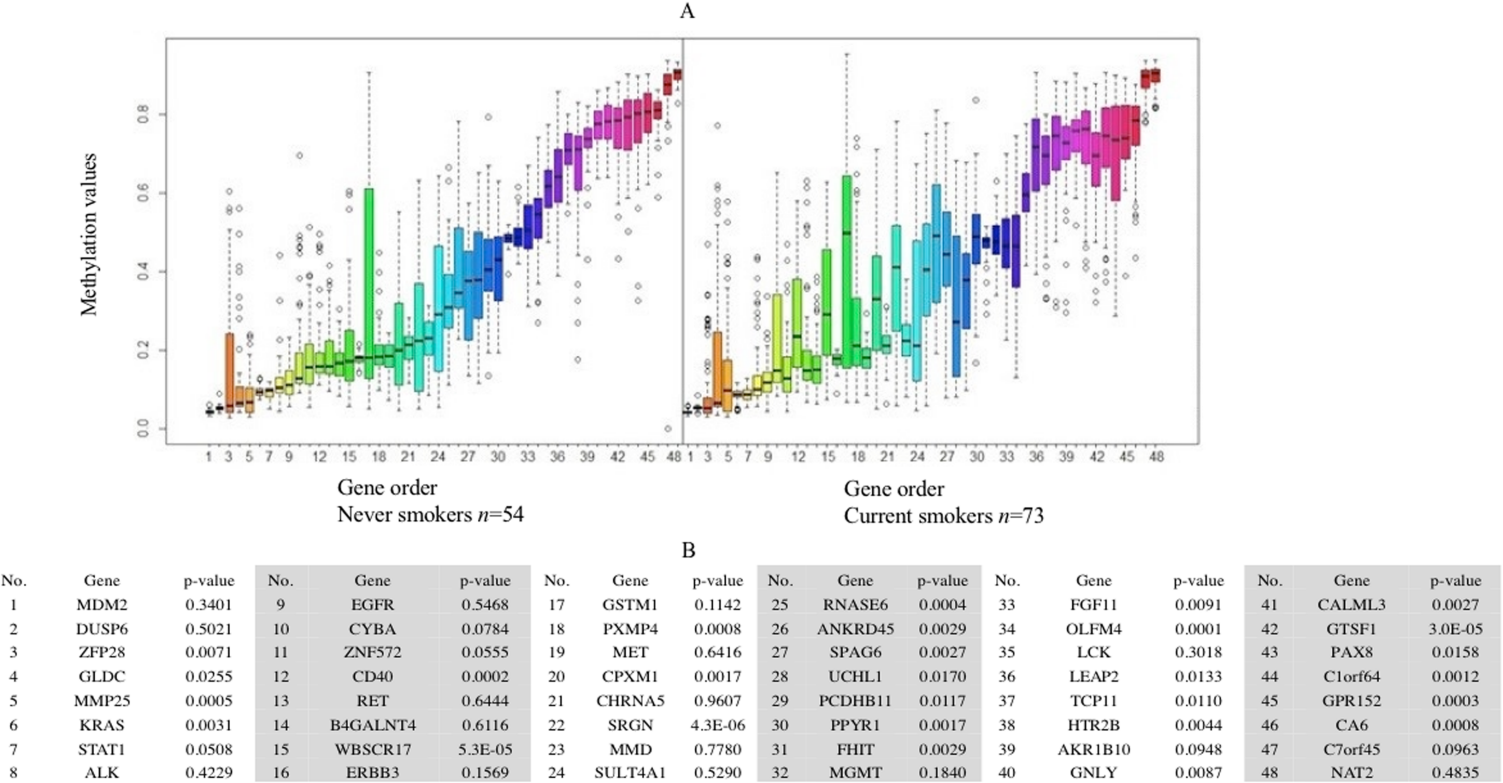
Boxplot of methylation values of ME signature genes in the TCGA dataset. (A) Boxplots of methylation values of ME signature genes in never smokers and current somkers of the TCGA dataset. Genes are listed in (B). Signature genes were sorted by the median values of their methylation levels in the nonsmokers.

Forty-eight genes were identified as ME signature genes (Table S3). ME levels in the signature genes of current smokers are more variable than those in nonsmokers (Fig. 3), especially for GLDC (4th), CYBA (10th), CD40 (12th), WBSCR17 (15th), PXMP4 (18th) and C1orf64 (44th).

**Figure 4:**
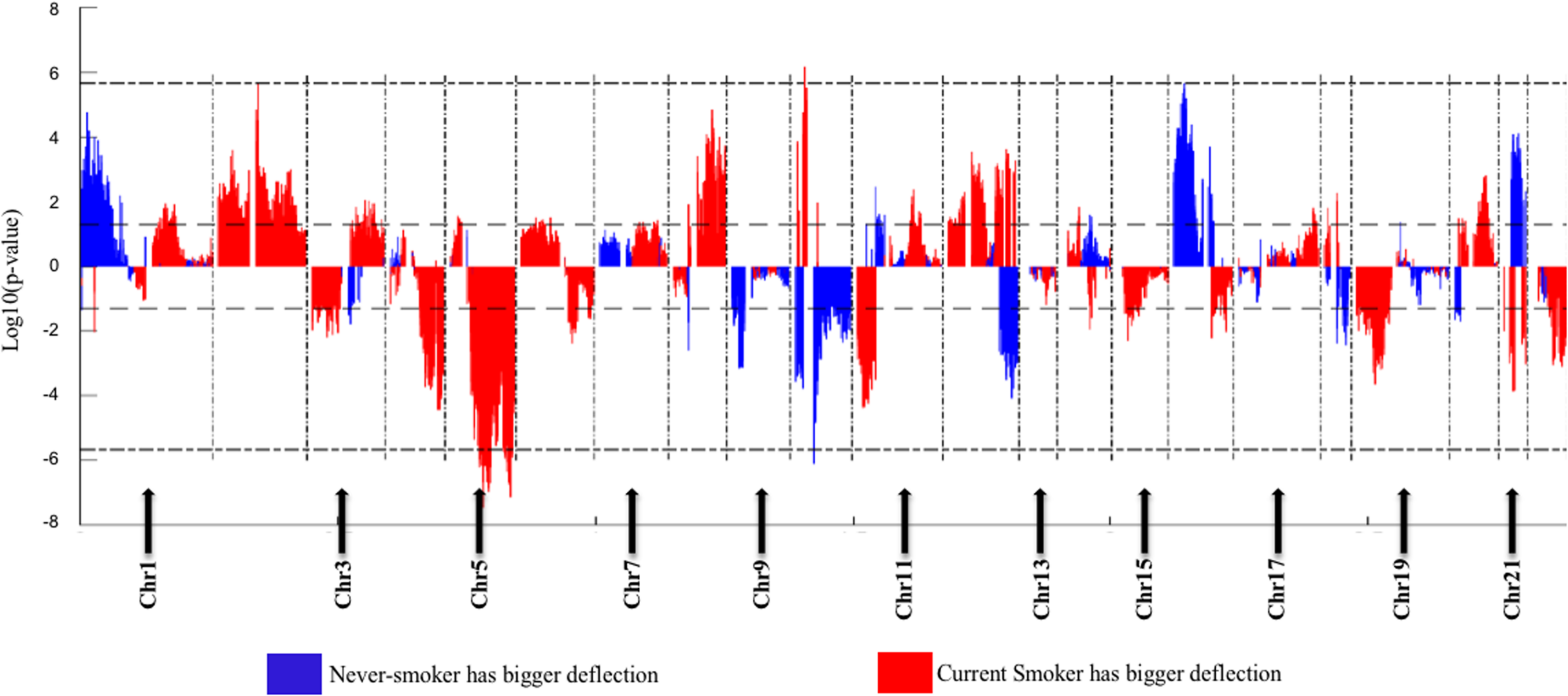
Overview of the differences of the genome CNV (deflection plots). Blue color indicates that the deflection (nonsmokers vs. current smokers) was greater for LUAD, whereas red color indicates that the deflection was greater for current smokers. Dashed horizontal lines are the cutoff lines according to the Bonferonni correction (for 23,494, −log10 (2.10E−6) = 5.68). The vertical dashed lines separate the data of each chromosome. A gap within the individual chromosome data indicates the location of the centrosome. For chromosomes 13, 14, 15, 21 and 22 only genes on the *q* arm were represented on the microarray.

The Deflection Plot shows that only 1% of CNV patterns in the whole genome are significantly different between current smokers and nonsmokers, after Bonferroni-correction (Fig. 4). This low percentage tells us that the difference in CNV patterns between current smokers and nonsmokers is minor.

**Figure 5:**
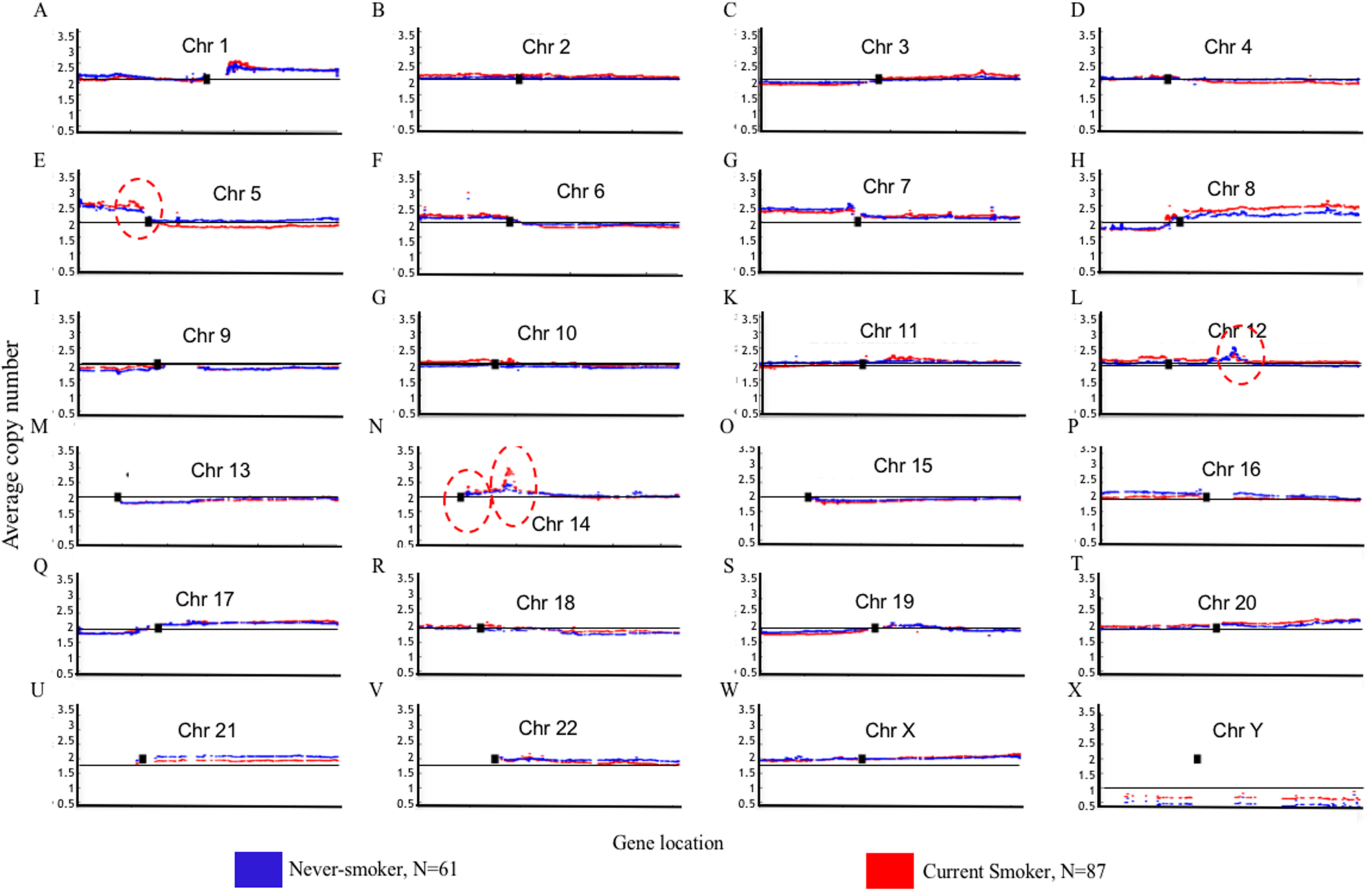
Overview of the genome mountain plot of CNV in current smokers and nonsmokers in TCGA LUAD samples. In subplots (A)–(X) each spot represents the median value of copy numbers of each gene in the corresponding group. The genes are sorted according to their locations. The space between two arms of each chromosome is the location of the corresponding centromere.

Genome Mountain plots of CNV patterns in current smokers and nonsmokers in TCGA LUAD samples also confirms that the difference between the CNV patterns in these two groups is minor (Fig. 5). There are only broadly mild negative variations between 5q, 16p and 21q. Additionally, CNV patterns on 8q, Chr12 and ChrY in current smokers are broadly higher than those in nonsmokers. Most importantly, considering that the normal value of ChrY should be one, CNVs on it in nonsmokers are much lower than those of current smokers. This may be related to the initiation or progression mechanism of LUAD in male nonsmokers.

Aside from these broad and minor differences between smokers and nonsmokers, there are four significant variations (highlighted with red circles in Fig. 5); their enlarged Mountain plots are shown in Figs. S1–S3, respectively.

Figure S1 shows the Mountain Plot of CNV patterns on Chr5 in current smokers and nonsmokers of LUAD in the TCGA dataset. There are seven genes on the peak of the focal amplicon located on 5p13.2: UGT3A1, UGT3A2, LMBRD2, MIR580, SKP2, NADK2 and RANBP3L. Among them, S-Phase Kinase Associated Protein 2 (SKP2) has been shown to play a critical role in cell cycle progression, especially at the G(1)-S transition, putatively through its control of several cell cycle regulator proteins (Zhu et al., 2004). Though the SKP2 gene is commonly overexpressed in lung cancer, we can see that it is more overexpressed in current smokers than it is in nonsmokers. On the contrary, Telomerase Reverse Transcriptase (TERT), another well-known gene for NSCLC (Kang et al., 2008), is on the peak of the focal amplicon at 5p15.33 of nonsmokers. The amplification of TERT in nonsmokers may play an important role in the initiation or progression of LUAD in never-smoking patients.

Figure S2 shows the focal amplicon at 12q15 whose peak gene is murine double minute 2 (MDM2). MDM2 is known as the negative regulator of the p53 pathway, which is a very important suppressor pathway for NSCLC (Hai et al., 2015; Javid et al., 2015). From its subplot, we can see that MDM2 is more amplified in female nonsmokers than it is in male nonsmokers. This may provide a clue about why there are comparatively more female never-smoking LUAD patients (Liu et al., 2006). MDM2 is identified as a GE, ME and CNV signature gene at the same time, which strongly indicates its importance in tobacco exposure classification as a never-smoking signature.

Figure S3 shows the focal amplicon at 14q13.3 whose peak gene is NK2 homeobox 1 (NKX2-1, which is known as a thyroid transcription factor 1 (TTF-1)). NKX2-1 is a key molecule in lung development. It is highly expressed in NSCLC, particularly in LUAD, where it is a diagnostic marker (Moises et al., 2017). Figure S3 shows that the average copy number of NKX2-1 in current smokers is much greater than it is in the nonsmokers.

The best classification results obtained by GE→tobacco, ME→tobacco and CNV→tobacco models for the TCGA, SPORE and EDRN datasets are shown in Table 2. We can see that using only 43 GE signature genes, the accuracy of tobacco exposure pattern classification is as high as 79.2% (sensitivity = 81.5%; specificity = 76.9%). The difference between specificity and sensitivity is only 4.6%. More convincingly, for the EDRN independent validation dataset, accuracy is 86.3% (sensitivity = 84.2%; specificity = 88.5%). The difference between specificity and sensitivity is only 4.3%. Meanwhile, for 48 ME signature genes, the accuracy of TCGA training samples is as high as 87.5%. The corresponding sensitivity and specificity are 87.2% and 87.8%, respectively. The difference between them is only 0.6%. For the EDRN independent validation dataset, accuracy is 76.4% and the difference between sensitivity and specificity (sensitivity = 80.2%; specificity = 73.6%) is 6.6%. Even for CNV→tobacco, the accuracies in the TCGA training and SPORE validation data are 77.1% and 77.3%, respectively. The differences between sensitivity and specificity in both datasets are 11.1% and 7.0%, respectively. These differences are slightly higher compared with what was obtained for the GE→tobacco and ME→tobacco models, but they are still acceptable.

**Table 2:** Classification results obtained by GE, ME and CNV signature values.

Data	Dataset	Numbers of signature genes	SN (%)	SP (%)	ACC (%)
GE	TCGA (training)	43	81.5	76.9	79.2
	EDRN (validation)		84.2	88.5	86.3
ME	TCGA (training)	48	87.2	87.8	87.5
	EDRN (validation)		80.2	73.6	76.4
CNV	TCGA (training)	75	82.2	71.1	77.1
	SPORE (validation)		80.3	73.3	77.3

Since the predictive performance for a classification model is more important than the modeling performance, the accuracy for data validation is correspondingly more important. From Table 2, we can see that the lowest prediction accuracy is obtained by the CNV→tobacco model while the highest is obtained by the GE→tobacco model, which means mRNA expression may have a closer relationship to tobacco exposure than CNV.

For GE, ME and CNV signature genes, we identified 7, 9 and 5 significant KEGG pathways respectively whose *p*-values are less than 0.05 (Tables S5–S7). Most of them are associated with cancer, such as NSCLC (hsa05223) and bladder cancer. Additionally, endocytosis and the ErbB signaling pathway are both important cancer-related pathways enriched by our signature genes. More importantly, the hsa05200 pathway derived from both ME and CNV signatures, which includes EGFR, KRAS, MDM2, STAT1 and other signature genes identified by us, is shown to be closely related to initiation and progression of cancer. These pathway analysis results strongly support the essentiality of our identified signature genes.

## Discussion

To overcome the overwhelming imbalance between the dimensions of samples and genes and the adverse influence of noise inherent in microarray/sequencing data, we proposed a novel integrated method to identify signature genes from thousands of genes in the whole genome. Considering factors such as statistical importance, biological function and the contribution to the tobacco exposure pattern classification model, comparatively important genes were first selected as candidate signature genes to reduce the gene dimension and the inherent noise. Then they were refined iteratively on the basis of the optimization of the classification performance. The gene set with the highest classification accuracy was identified as the final set of signature genes. Figure S4 shows examples of the Receiver Operating Characteristic (ROC) curves of model training and validating using GE data of TCGA and EDRN datasets.

As mentioned above, we can see that the GE→tobacco model has the highest prediction accuracy for the EDRN data (Table 2). It means the GE→tobacco model also has the highest predictive performance for new samples. This may be due to the fact that expression values of genes are closely related with tobacco exposure. It may be also due to the fact that the quality of the data in both the TCGA and EDRN is good enough. On the contrary, the platform for EDRN ME data is only 27 K, much lower than the measuring precision of the 450 K platform used for TCGA ME data. Meanwhile, the quality of SPORE CNV data is also much lower than that of TCGA CNV data. Irrespective of whether the quality of data is good or not, the prediction accuracies of all models exceed 76%. Thus, the robustness of our tobacco classification models and the representativeness of signature genes are strongly indicated.

Figure S5 shows the Venn diagram of the signature genes identified by the GE, ME and CNV analyses. There are 13 genes identified as both GE and ME signature genes and 12 genes identified as both ME and CNV signature genes. Most importantly, five genes are identified as GE, ME and CNV signature genes: MDM2, GSTM1, MGMT, RET and ALK (anaplastic lymphoma kinase).

- MDM2 (an oncogene (Wang et al., 2019)) is located at the peak of the focal amplification at 12q15 as shown in Fig. S2. The start and end genes of this focal amplification are LOC102724421 and TPH2. MDM2 is known as a negative regulator of the p53 pathway, which is a very important suppressor pathway for NSCLC (Hai et al., 2015; Javid et al., 2015). Since MDM2 can suppress the suppressor gene TP53 of NSCLC, the amplified MDM2 in nonsmokers may play a very important role for the initiation or progression of LUAD of nonsmokers. From the subplot of Fig. S2, we also can see that MDM2 is more amplified in female nonsmokers than it is in male nonsmokers. This may explain why more female nonsmokers tend to suffer LUAD (Liu et al., 2006).
- Glutathione S transferase mu 1 (GSTM1) gene has been shown to be associated with lung cancer risk, and the GSTM1 enzyme plays a vital role in the detoxification pathway and protection against toxic insults (Yu et al., 2018). There are reports that GSTM1 has no relevant modifying effect on lung cancer risk and cumulative smoking dose (Schneider et al., 2004; Pisani et al., 2006). But there are also reports the GSTM1 genotype both alone and in combination with the GSTP1 genotype alters the risk of developing lung cancer among nonsmokers (Wenzlaff et al., 2005). The Pearson coefficient value between its gene expression values and ME values is −0.877 (shown in Fig. S6). This means that the gene expression values are strongly negatively related with ME levels. This was consistent with the results of Selamat et al. (2011). Its hypermethylation in current smokers resulted in the loss of gene expression, which led to less expression of the gstm1-1 enzyme and the GSTM3-3 enzyme. Therefore, the capability of detoxification could not be normal which might increase tobacco smoking carcinogens.
- O(6)-methylguanine-DNA methyltransferase (MGMT) promoter ME has been demonstrated to be associated with increased occurrence of p53 mutation including the G:C→A:T transition and other p53 mutation patterns in lung cancer, especially among nonsmokers (Liu et al., 2006; Wu et al., 2008).
- Genetic alterations, including mutation of the epidermal growth factor receptor (or v-Ki-ras2 kirsten rat sarcoma viral oncogene homolog) and fusion of ALK, RET proto-oncogene (RET), or v-ros UR2 sarcoma virus oncogene homolog 1 (ROS1), occur in NSCLCs, and these oncogenic drivers are important biomarkers for targeted therapies (Furugaki et al., 2019; Tan et al., 2018).
- ALK (EML4-ALK is a well-known fusion oncogene (Lee et al., 2019)) translocations are more frequent in nonsmokers’ than in smokers’ lung cancer (Torres-Duran et al., 2017). Therefore, it is a reasonable signature gene for nonsmokers. The treatment options for EGFR-mutated and ALK-rearranged NSCLCs are distinctly different from those of lung cancer that lacks actionable mutations (Chalmers et al., 2019).

EGFR is one of the major mutation drivers frequently present in LUADs (Kim et al., 2018). Our analysis identified it as both GE and ME signatures. This means that beside the mutation status of EGFR, its expression values and ME levels are also very important to tobacco exposure classification.

More importantly, several other GE signatures have been confirmed to be up-regulated, for example, up-regulation of UCHL1 at the protein level was observed with immunohistochemical analysis of bronchial biopsies of smokers compared with nonsmokers and its overexpression in chronic smokers may represent an early event in the complex transformation from normal epithelium to overt malignancy (Carolan et al., 2006); and CYP24A1 was found to be significantly up-regulated in NSCLC patients by Kim et al. (2007).

Seventy-five genes were identified as tobacco-related CNV signature genes (Table S4). Ten of these (e.g., KRAS, MDM2 and so on) have been identified previously. Among these 75 genes, 13 genes are located on 8q which is a broad amplification of smokers compared with nonsmokers. In particular, 12 of these 13 genes are located on 8q21-24. According to the biological mechanism, CNVs of adjacent genes are closely related to each other. This may be the reason that why potential CNV signatures are close to each other and why it takes more CNV signature genes to distinguish smokers from nonsmokers.

Among 12 of these 13 genes located on 8q21-24: PVT1 represents a long non-coding RNA locus that has been identified as a candidate oncogene. Increased copy number and overexpression of this gene are associated with many types of cancers including breast and ovarian cancers, acute myeloid leukemia and Hodgkin lymphoma (Bhide et al., 2010; Kodaira et al., 2009; Saibishkumar et al., 2007). ZFAT encodes a protein that likely binds DNA and functions as a transcriptional regulator involved in apoptosis and cell survival (Ishikura et al., 2015). TRAPPC9 encodes a protein that likely plays a role in NF-kappa Tumor Protein D52 is showed to be associated with Lung Squamous Cell Carcinoma. Among its related pathways of Copine 3 are Innate Immune System and Metabolism. PLEKHF2 may play a role in early endosome fusion upstream of RAB5, hence regulating receptor trafficking and fluid-phase transport. Enhances cellular sensitivity to TNF-induced apoptosis (Laifenfeld et al., 2007).

Apart from these potential CNV signatures, NKX2-1 (TTF1), located on 14q13.3, is also a very important gene as we mentioned above. The contribution of its CNV to the classification model is however not as important as the CNVs of the 75 CNV signature genes. This may be due to the fact that every data-driven method has its limitations. It could not overcome the strong relationship among adjacent genes in CNV patterns. It may be also due to NKX2-1 working through other signature genes. As a result, NKX2-1 is buried by other genes’ CNV contribution. The normal CNV values of genes on ChrY are only half of the values of genes located on autosomes. For the same reason, therefore, no genes on ChrY were identified as signatures since their absolute CNV values were not big enough to be uncovered. This is why, aside from data-driven signature gene identification, genome-wide CNV pattern analysis is still very important.

Since the patterns of driven gene were different between the Caucasian and the Asian. The Caucasian were the predominant one in the used databases, especially in the TCGA training data. Therefore, the results obtained by our study are mainly regarding to the Caucasian.

## Conclusion

To accelerate the development of precision medicine for LUAD with different tobacco exposure, a novel method was proposed to select potential tobacco-related signature genes out of approximately 20,000 genes. This method integrates statistical selection, experimental selection and iteratively contribution selection methods to identify signature genes to distinguish samples of smokers from nonsmokers. Three sets of tobacco-related signature genes are identified through this proposed integrative method. The excellent classification performance, the molecular mechanism analysis and the KEGG pathway analysis strongly support their important roles in cancer initiation and progression. Additionally, the broad deletion on ChrY and the amplicon on TERT in nonsmokers, especially the amplicon of MDM2 in female nonsmokers may provide clues for the initiation and progression of LUAD in nonsmokers of different genders.

## Supplemental Information

10.7717/peerj.8349/supp-1 Supplemental Information 1 Supplemental Tables.

10.7717/peerj.8349/supp-2 Supplemental Information 2 Supplemental Figures.

## Additional Information and Declarations

### Data Availability

The following information was supplied regarding data availability: TCGA data is Available at https://portal.gdc.cancer.gov/projects. The level three GE, CNV and ME data of LUAD patients were used in our study. EDRN data is Available at http://www.ncbi.nlm.nih.gov/geo/ with the Accession Number GSE32867 (GSE32867–GPL6884 for expression and GSE32867–GPL8490 for ME). SPORE data is Available at http://www.ncbi.nlm.nih.gov/geo/ with the Accession Number GSE74948.
