# Supplementary material for: Multiple genome pattern analysis and signature gene identification for the Caucasian lung adenocarcinoma patients with different tobacco exposure patterns
Source: PeerJ. 2020 Jan 30;8:e8349. doi: 10.7717/peerj.8349 (PMC6995662; doi:10.7717/peerj.8349)
Supplement: Supplemental Information 1 [file peerj-08-8349-s001.pptx]

## Slide 1
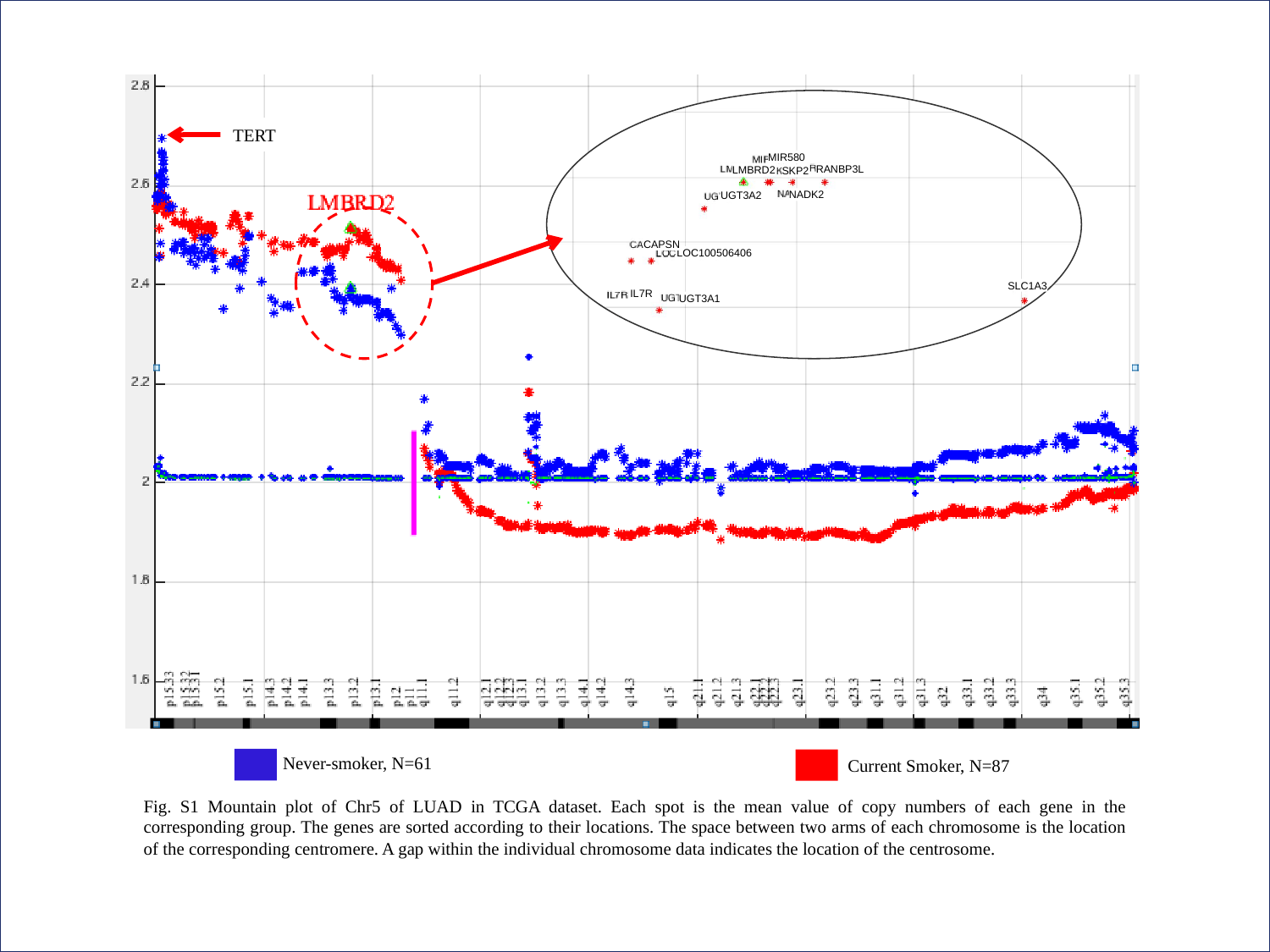

TERT
MIR580
RANBP3L
LMBRD2
SKP2
NADK2
UGT3A2
CAPSN
LOC100506406
SLC1A3
IL7R
UGT3A1
Never-smoker, N=61
Current Smoker, N=87
Fig. S1 Mountain plot of Chr5 of LUAD in TCGA dataset. Each spot is the mean value of copy numbers of each gene in the corresponding group. The genes are sorted according to their locations. The space between two arms of each chromosome is the location of the corresponding centromere. A gap within the individual chromosome data indicates the location of the centrosome.

## Slide 2
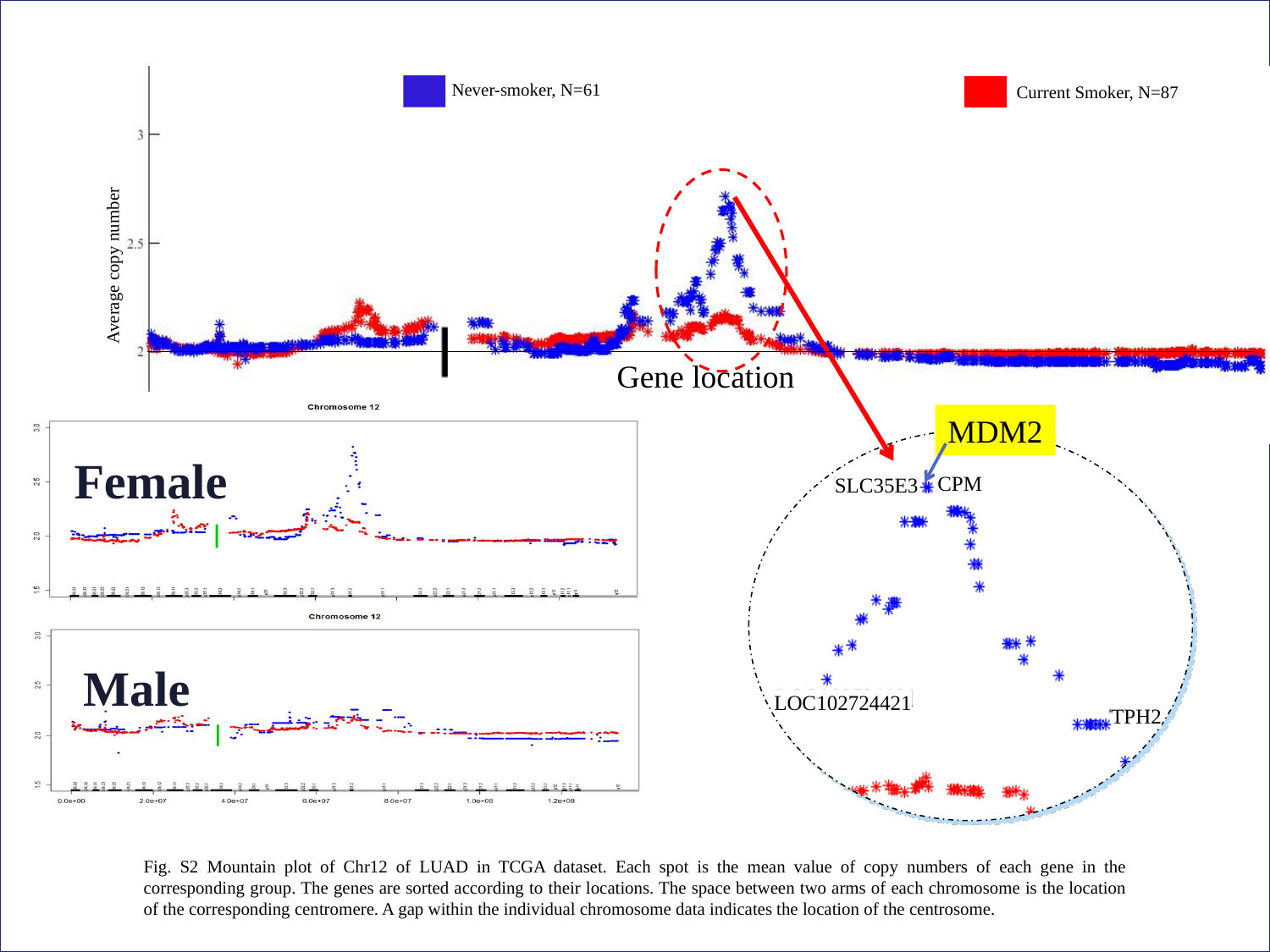

Never-smoker, N=61
Current Smoker, N=87
Average copy number
Gene location
MDM2
Female
CPM
SLC35E3
Male
LOC102724421
TPH2
Fig. S2 Mountain plot of Chr12 of LUAD in TCGA dataset. Each spot is the mean value of copy numbers of each gene in the corresponding group. The genes are sorted according to their locations. The space between two arms of each chromosome is the location of the corresponding centromere. A gap within the individual chromosome data indicates the location of the centrosome.

## Slide 3
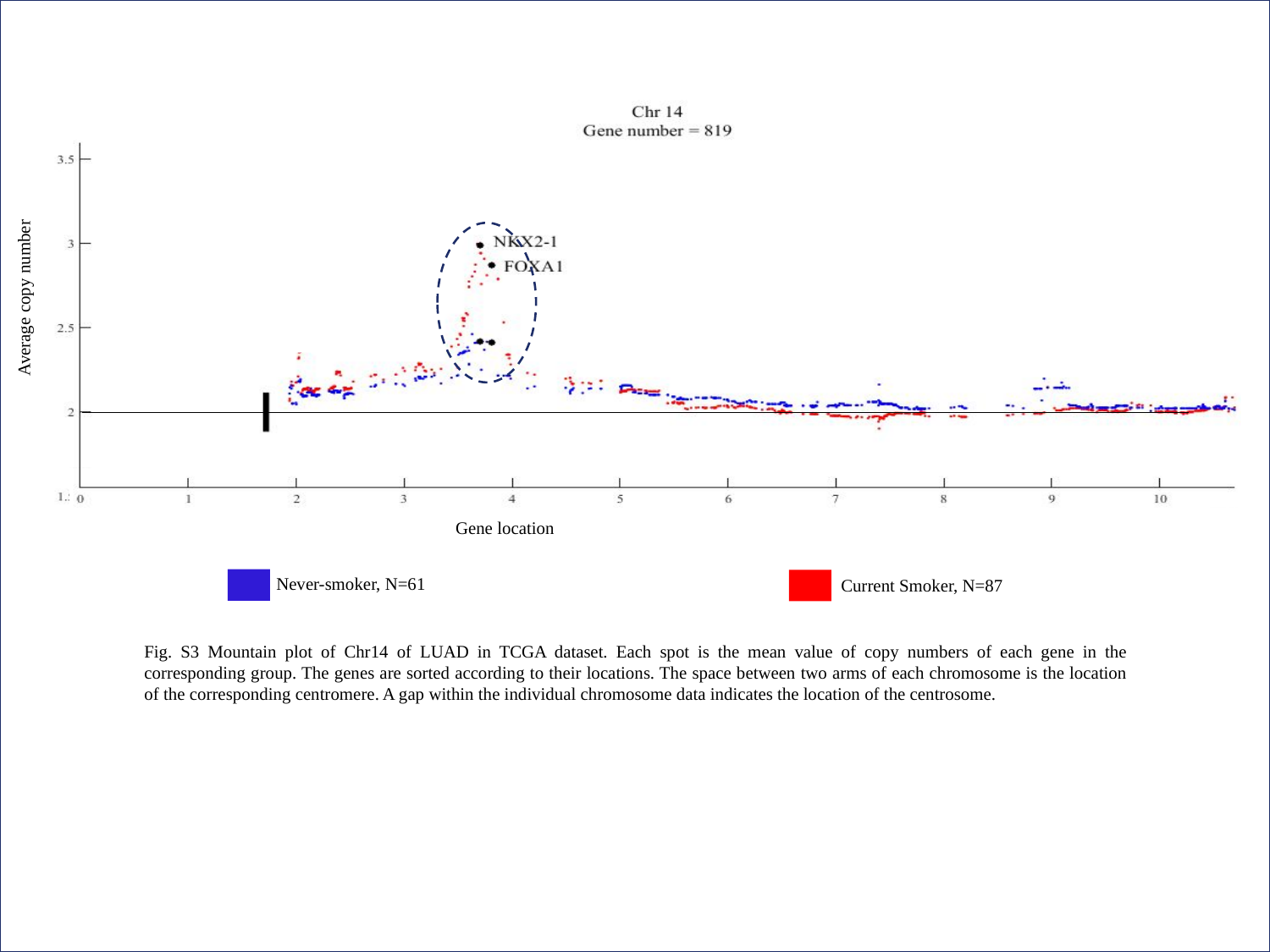

Average copy number
Gene location
Never-smoker, N=61
Current Smoker, N=87
Fig. S3 Mountain plot of Chr14 of LUAD in TCGA dataset. Each spot is the mean value of copy numbers of each gene in the corresponding group. The genes are sorted according to their locations. The space between two arms of each chromosome is the location of the corresponding centromere. A gap within the individual chromosome data indicates the location of the centrosome.

## Slide 4
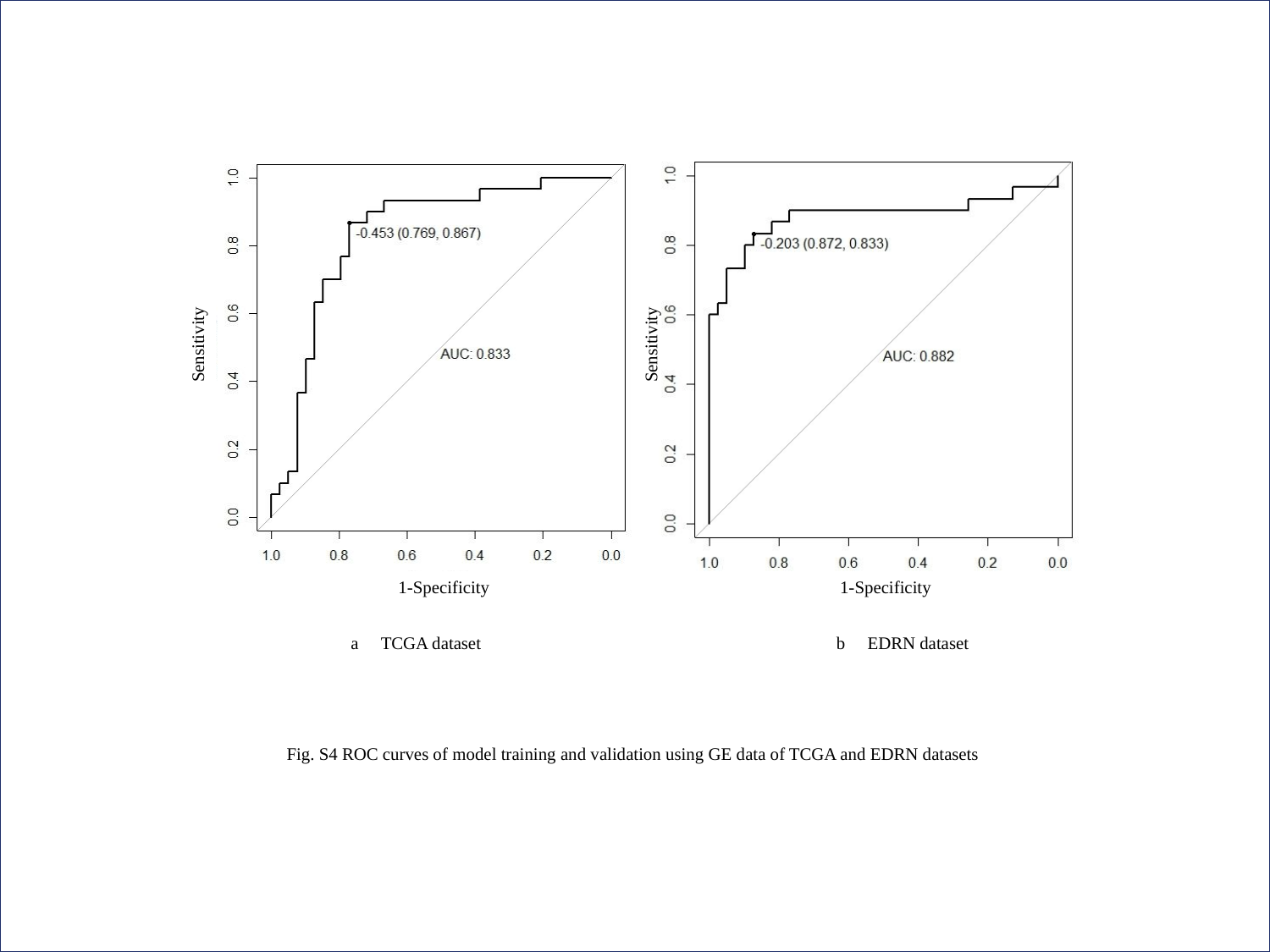

Sensitivity
Sensitivity
1-Specificity
1-Specificity
（a）TCGA dataset （b）EDRN dataset
Fig. S4 ROC curves of model training and validation using GE data of TCGA and EDRN datasets

## Slide 5
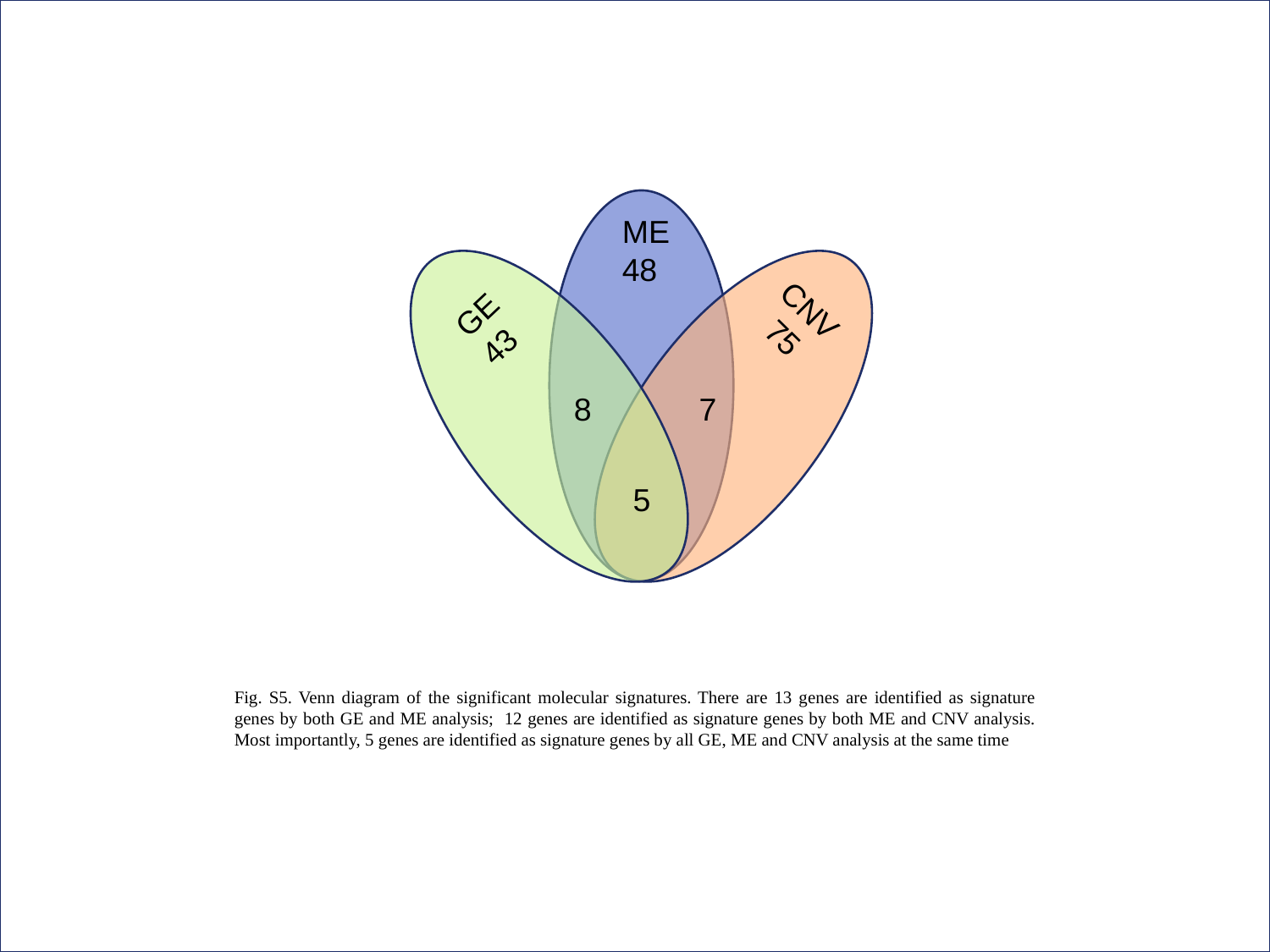

ME
48
CNV
75
GE
43
8
7
5
Fig. S5. Venn diagram of the significant molecular signatures. There are 13 genes are identified as signature genes by both GE and ME analysis; 12 genes are identified as signature genes by both ME and CNV analysis. Most importantly, 5 genes are identified as signature genes by all GE, ME and CNV analysis at the same time

## Slide 6
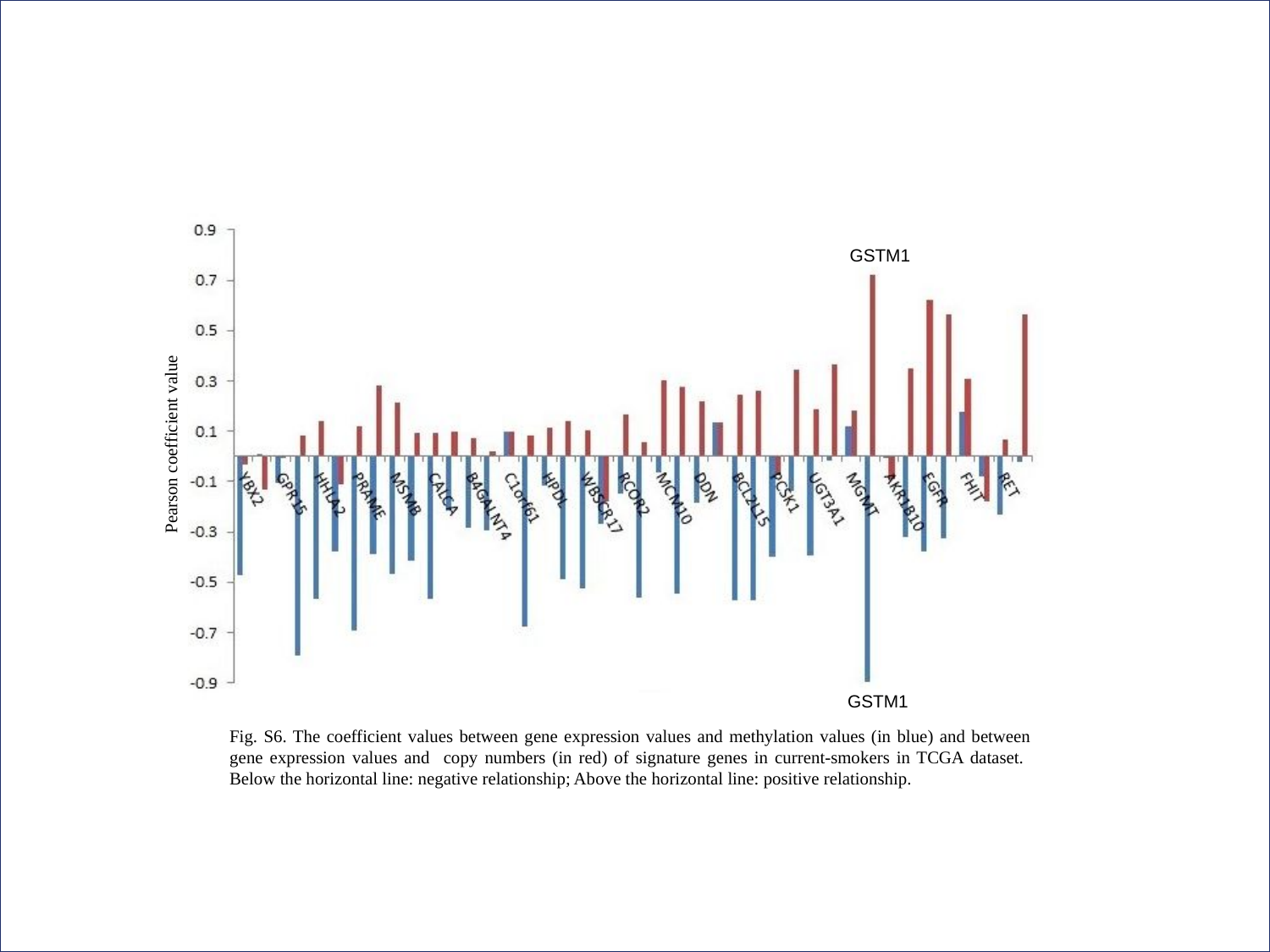

GSTM1
Pearson coefficient value
 GSTM1
Fig. S6. The coefficient values between gene expression values and methylation values (in blue) and between gene expression values and copy numbers (in red) of signature genes in current-smokers in TCGA dataset.
Below the horizontal line: negative relationship; Above the horizontal line: positive relationship.
