# Supplementary material for: Multiple genome pattern analysis and signature gene identification for the Caucasian lung adenocarcinoma patients with different tobacco exposure patterns
Source: PeerJ. 2020 Jan 30;8:e8349. doi: 10.7717/peerj.8349 (PMC6995662; doi:10.7717/peerj.8349)
Supplement: Supplemental Information 2 [file peerj-08-8349-s002.docx]

**Supplementary materials:**

**Multiple genome pattern analysis and signature gene identification for the Caucasian lung adenocarcinoma patients with different tobacco exposure patterns**

Yan-mei Dong^1＃^, Li-da Qin^1＃^, Yi-fan Tong^1^, Qi-en He^1^, Ling Wang^2^*, Kai Song^1^*

^1^ School of Chemical Engineering and Technology, Tianjin University, Tianjin, China

^2^ The First Affiliated Hospital Oncology of Dalian Medical University, Dalian, Liaoning, China

Correspondence should be addressed to: Kai Song

Email: ksong@tju.edu.cn

Address: 135 Yaguan Rd, Jinnan District, Tianjin, China, 300350

Tel: +86 (189)-2031-7821

Ling Wang

The First Affiliated Hospital Oncology of Dalian Medical University, Dalian, Liaoning, China

Email: [516245530@qq.com](mailto:516245530@qq.com)

# They contributed equally to this work

**Materials and Methods**

**The measurement of the classification performance**

To evaluate the performance of the classification model, the prediction accuracy, specificity and sensitivity were calculated as the following equations:

$$\text{ ACC=}\frac{\text{TP+TN}}{\text{TP+TN+FP+FN}} \left( 1 \right)$$

$$\text{ SN=}\frac{\text{TP}}{\text{TP+FN}} (2)$$

$$\text{ SP=}\frac{\text{TN}}{\text{TN+FP}} (3)$$

In general:

- True positive (TP): correctly classified as positive samples
- False positive (FP): incorrectly classified as positive samples
- True negative (TN): correctly classified as negative samples
- False negative (FN): incorrectly classified as negative samples

In our study, nonsmokers were used as the positive samples and current-smokers were used as the negative samples. Sensitivity, SN, is the proportion of nonsmokers that are correctly classified as the nonsmokers. Specificity, SP, is the proportion of current-smokers which are correctly classified as the current-smokers. Accuracy, ACC, is the proportion of samples which are correctly classified.

There are four types of smoking history in TCGA dataset: current-smoker, reformed less than 15 years, reformed longer than 15 years and lifelong nonsmoker. On the contrary, there are only three types of smoking history in EDRN: current-smoker, former-smoker and lifelong nonsmoker. For SPORE data, only ever or never smoking status is available. Due to the different types in these datasets and the arbitrary cutoff and definition of smoking history, in order to identify the most significant signature genes, only typical samples *i.e.* current-smokers and lifelong nonsmokers in TCGA were used as training samples in our study. EDRN (current-smokers and lifelong nonsmokers) and SPORE (ever/never smokers) data were used as independent validation samples.

**Table S1. The list of experimental proved important genes**

| No. | Gene | Location | No. | Gene | Location | No. | Gene | Location | No. | Gene | Location |
| --- | --- | --- | --- | --- | --- | --- | --- | --- | --- | --- | --- |
| 1 | GSTM1[[1](#_ENREF_1)] | 1p13.3 | 7 | EGFR[[2](#_ENREF_2)] | 7p12 | 13 | MGMT[[3](#_ENREF_3)] | 10q26 | 19 | MEK1[[4](#_ENREF_4)] | 15q22.1-q22.33 |
| 2 | LCK[[5](#_ENREF_5)] | 1p34.3 | 8 | MET[[6](#_ENREF_6)] | 7q31 | 14 | KRAS[[7](#_ENREF_7)] | 12p12.1 | 20 | CHRNA5[[8](#_ENREF_8)] | 15q24 |
| 3 | ALK[[2](#_ENREF_2)] | 2p23 | 9 | AKR1B10[[9](#_ENREF_9)] | 7q33 | 15 | ERBB3[[10](#_ENREF_10)] | 12q13 | 21 | MMD[[11](#_ENREF_11)] | 17q |
| 4 | STAT1[[12](#_ENREF_12)] | 2q32.2 | 10 | BRAF[[13](#_ENREF_13)] | 7q34 | 16 | MDM2[[14](#_ENREF_14)] | 12q14.3-q15 | 22 | XRCC1[[15](#_ENREF_15)] | 19q13.2 |
| 5 | FHIT[[16](#_ENREF_16)] | 3p14.2 | 11 | NAT2[[17](#_ENREF_17)] | 8p22 | 17 | DUSP6[[18](#_ENREF_18)] | 12q22-q23 | 23 | ERCC2[[15](#_ENREF_15)] | 19q13.3 |
| 6 | ROS-1[[19](#_ENREF_19)] | 6q22 | 12 | RET[[19](#_ENREF_19)] | 10q11.2 | 18 | OLFM4[[20](#_ENREF_20)] | 13q21.1 | 24 | RASSF2[[21](#_ENREF_21)] | 20p13 |

**Table S2. The list of 43 GE signature genes^*^**

| No. | Gene | Location | Median Value | |
| --- | --- | --- | --- | --- |
|  |  |  | Never Smokers | Current Smokers |
| 1 | BCL2L15 | 1p13.2 | 8.713 | 7.265 |
| 2 | GSTM1 | 1p13.3 | 7.727 | 1.546 |
| 3 | ZYG11A | 1p32.3 | 4.147 | 5.557 |
| 4 | HPDL | 1p34.1 | 3.255 | 4.998 |
| 5 | C1orf61 | 1q22 | 1.352 | 2.993 |
| 6 | FAM72A | 1q32.1 | 3.178 | 4.962 |
| 7 | GNG4 | 1q42.3 | 4.048 | 5.319 |
| 8 | ALK | 2p23 | 2.317 | 2.059 |
| 9 | CGREF1 | 2p23.3 | 5.100 | 6.926 |
| 10 | FHIT | 3p14.2 | 5.084 | 5.054 |
| 11 | GPR15 | 3q11.2-q13.1 | 0.922 | 3.986 |
| 12 | HHLA2 | 3q13.13 | 7.629 | 4.218 |
| 13 | UCHL1 | 4p14 | 7.461 | 9.535 |
| 14 | FGB | 4q28 | 1.814 | 5.777 |
| 15 | FGG | 4q28 | 7.516 | 9.828 |
| 16 | HPGD | 4q34-q35 | 10.935 | 9.535 |
| 17 | UGT3A1 | 5p13.2 | 0 | 0 |
| 18 | PCSK1 | 5q15-q21 | 2.248 | 3.727 |
| 19 | EGFR | 7p12 | 10.409 | 10.048 |
| 20 | WBSCR17 | 7q11.23 | 6.424 | 4.661 |
| 21 | AKR1B10 | 7q33 | 4.866 | 4.279 |
| 22 | BARX1 | 9q12 | 2.003 | 4.068 |
| 23 | MCM10 | 10p13 | 5.636 | 7.641 |
| 24 | MSMB | 10q11.2 | 3.229 | 5.218 |
| 25 | RET | 10q11.2 | 4.650 | 4.442 |
| 26 | MGMT | 10q26 | 8.089 | 8.360 |
| 27 | CALCA | 11p15.2 | 0.700 | 1.562 |
| 28 | B4GALNT4 | 11p15.5 | 4.570 | 7.014 |
| 29 | RCOR2 | 11q13.1 | 4.707 | 6.272 |
| 30 | KRAS | 12p12.1 | 10.220 | 10.348 |
| 31 | ERBB3 | 12q13 | 12.093 | 12.168 |
| 32 | DDN | 12q13.12 | 1.814 | 3.531 |
| 33 | MDM2 | 12q14.3-q15 | 10.914 | 10.768 |
| 34 | POU4F1 | 13q31.1 | 0.499 | 3.241 |
| 35 | ERN2 | 16p12.2 | 7.562 | 5.426 |
| 36 | YBX2 | 17p13.1 | 2.125 | 5.960 |
| 37 | DLL3 | 19q13 | 0.903 | 3.946 |
| 38 | LGALS4 | 19q13.2 | 5.170 | 3.407 |
| 39 | PLUNC | 20q11.2 | 6.367 | 8.314 |
| 40 | CYP24A1 | 20q13 | 6.781 | 9.092 |
| 41 | MYBL2 | 20q13.1 | 8.539 | 10.085 |
| 42 | UBE2C | 20q13.12 | 7.885 | 9.494 |
| 43 | PRAME | 22q11.22 | 2.856 | 7.054 |

* genes are sorted according to their contribution to the classification model.

**Table S3. The list of 48 methylation signature genes^*^**

| No. | Gene | Location | Median Value | |
| --- | --- | --- | --- | --- |
|  |  |  | Never Smokers | Current Smokers |
| 1 | GSTM1 | 1p13.3 | 0.354 | 0.430 |
| 2 | LCK | 1p34.3 | 0.616 | 0.602 |
| 3 | C1orf64 | 1p36.13 | 0.771 | 0.690 |
| 4 | CA6 | 1p36.2 | 0.804 | 0.759 |
| 5 | ANKRD45 | 1q25.1 | 0.404 | 0.483 |
| 6 | GNLY | 2p11.2 | 0.775 | 0.746 |
| 7 | ALK | 2p23 | 0.121 | 0.131 |
| 8 | PAX8 | 2q12-q14 | 0.773 | 0.725 |
| 9 | STAT1 | 2q32.2 | 0.093 | 0.087 |
| 10 | HTR2B | 2q36.3-q37.1 | 0.656 | 0.724 |
| 11 | FHIT | 3p14.2 | 0.484 | 0.469 |
| 12 | UCHL1 | 4p14 | 0.383 | 0.314 |
| 13 | PCDHB11 | 5q31 | 0.416 | 0.359 |
| 14 | LEAP2 | 5q31.1 | 0.645 | 0.695 |
| 15 | TCP11 | 6p21.3-p21.2 | 0.702 | 0.662 |
| 16 | EGFR | 7p12 | 0.121 | 0.126 |
| 17 | WBSCR17 | 7q11.23 | 0.212 | 0.320 |
| 18 | MET | 7q31 | 0.182 | 0.179 |
| 19 | C7orf45 | 7q32.2 | 0.857 | 0.887 |
| 20 | AKR1B10 | 7q33 | 0.733 | 0.708 |
| 21 | NAT2 | 8p22 | 0.901 | 0.898 |
| 22 | ZNF572 | 8q24.13 | 0.177 | 0.145 |
| 23 | GLDC | 9p22 | 0.116 | 0.181 |
| 24 | SPAG6 | 10p12.2 | 0.358 | 0.434 |
| 25 | CALML3 | 10p15.1 | 0.775 | 0.730 |
| 26 | PPYR1 | 10q11.2 | 0.415 | 0.480 |
| 27 | RET | 10q11.2 | 0.187 | 0.195 |
| 28 | SRGN | 10q22.1 | 0.248 | 0.398 |
| 29 | MGMT | 10q26 | 0.493 | 0.481 |
| 30 | B4GALNT4 | 11p15.5 | 0.169 | 0.164 |
| 31 | GPR152 | 11q13.1 | 0.799 | 0.746 |
| 32 | KRAS | 12p12.1 | 0.094 | 0.087 |
| 33 | ERBB3 | 12q13 | 0.178 | 0.174 |
| 34 | GTSF1 | 12q13.2 | 0.757 | 0.677 |
| 35 | MDM2 | 12q14.3-q15 | 0.043 | 0.042 |
| 36 | DUSP6 | 12q22-q23 | 0.052 | 0.053 |
| 37 | OLFM4 | 13q21.1 | 0.529 | 0.451 |
| 38 | RNASE6 | 14q11.2 | 0.325 | 0.414 |
| 39 | CHRNA5 | 15q24 | 0.209 | 0.209 |
| 40 | MMP25 | 16p13.3 | 0.081 | 0.139 |
| 41 | CYBA | 16q24 | 0.190 | 0.237 |
| 42 | FGF11 | 17p13.1 | 0.507 | 0.465 |
| 43 | MMD | 17q | 0.227 | 0.223 |
| 44 | ZFP28 | 19q13.43 | 0.165 | 0.091 |
| 45 | CPXM1 | 20p13-p12.3 | 0.233 | 0.319 |
| 46 | PXMP4 | 20q11.22 | 0.195 | 0.266 |
| 47 | CD40 | 20q12-q13.2 | 0.192 | 0.268 |
| 48 | SULT4A1 | 22q13.2-q13.31 | 0.311 | 0.290 |

* genes are sorted according to their contribution to the classification model.

**Table S4. The list of 75 CNV signature genes^*^**

| Gene | Location | Current smoker | Never smoker | Gene | Location | Current smoker | Never smoker |
| --- | --- | --- | --- | --- | --- | --- | --- |
| RET | 10q11.2 | 2.0441 | 1.9317 | LCA5L | 21q22.2 | 1.9687 | 2.1224 |
| ALK | 2p23 | 2.101 | 2.0218 | HMGN1 | 21q22.3\|21q22.2 | 1.9695 | 2.1224 |
| LCK | 1p34.3 | 2.0082 | 2.1125 | WRB | 21q22.3 | 1.9677 | 2.1224 |
| RASSF2 | 20p13 | 2.0519 | 1.9471 | EFCAB6 | 22q13.1-q13.33 | 1.8891 | 1.9791 |
| STAT1 | 2q32.2 | 2.0906 | 2.037 | GDI2 | 10p15 | 2.0514 | 1.9339 |
| KRAS | 12p12.1 | 2.171 | 2.0627 | ARFGAP3 | 22q13.2-q13.3 | 1.8912 | 1.9794 |
| MGMT | 10q26 | 1.9714 | 1.8937 | PACSIN2 | 22q13.2 | 1.8893 | 1.9794 |
| MET | 7q31 | 2.2231 | 2.1386 | GNB1 | 1p36.33 | 2.0063 | 2.1228 |
| GSTM1 | 1p13.3 | 2.0859 | 2.1693 | DYRK1A | 21q22.13 | 1.9692 | 2.0907 |
| MDM2 | 12q14.3-q15 | 2.2075 | 2.5284 | DSCR3 | 21q22.2 | 1.9693 | 2.0907 |
| TSPAN17 | 5q35.3 | 1.9796 | 2.1148 | CPNE3 | 8q21.3 | 2.3434 | 2.1682 |
| UIMC1 | 5q35.2 | 1.9786 | 2.1148 | CHRAC1 | 8q24.3 | 2.4062 | 2.2497 |
| DDX41 | 5q35.3 | 1.9759 | 2.1023 | GTPBP4 | 10p15-p14 | 2.0379 | 1.9294 |
| PRELID1 | 5q35.3 | 1.9784 | 2.1013 | TRAPPC9 | 8q24.3 | 2.3727 | 2.2497 |
| ATG12 | 5q21-q22 | 1.8957 | 2.0293 | ATAD2 | 8q24.13 | 2.4438 | 2.2841 |
| COX7C | 5q14 | 1.895 | 2.0697 | STAU1 | 20q13.1 | 2.1446 | 2.062 |
| PGGT1B | 5q22.3 | 1.8994 | 2.0295 | OSTM1 | 6q21 | 1.8006 | 1.9566 |
| FEM1C | 5q22 | 1.9011 | 2.0293 | SEC63 | 6q21 | 1.8022 | 1.9566 |
| TAF7 | 5q31 | 1.9261 | 2.0329 | CD164 | 6q21 | 1.8044 | 1.9585 |
| DNAJA3 | 16p13.3 | 2.0561 | 2.192 | FIG4 | 6q21 | 1.8035 | 1.9585 |
| NMRAL1 | 16p13.3 | 2.0561 | 2.192 | SMPD2 | 6q21 | 1.8072 | 1.9585 |
| TMEM167A | 5q14.2 | 1.9027 | 2.0587 | ZBTB24 | 6q21 | 1.8067 | 1.9585 |
| MGRN1 | 16p13.3 | 2.0585 | 2.2007 | IMPACT | 18q11.2-q12.1 | 2.0188 | 1.9105 |
| RAB18 | 10p12.1 | 2.0792 | 1.9404 | ZFP64 | 20q13.2 | 2.1829 | 2.1053 |
| ABI1 | 10p11.2 | 2.0724 | 1.9398 | MRPS30 | 5q11 | 2.4329 | 2.3092 |
| YME1L1 | 10p14 | 2.0757 | 1.9398 | NUP155 | 5p13.1 | 2.5062 | 2.3741 |
| WAC | 10p12.1 | 2.0805 | 1.9404 | PANK4 | 1p36.32 | 2.0142 | 2.1231 |
| UBR5 | 8q22 | 2.3961 | 2.1848 | PEX10 | 1p36.32 | 2.0127 | 2.1231 |
| N4BP1 | 16q12.1 | 2.0035 | 2.1744 | TMEM68 | 8q12.1 | 2.2637 | 2.2064 |
| ICMT | 1p36.21 | 1.998 | 2.1299 | PVT1 | 8q24 | 2.5434 | 2.3937 |
| CAMTA1 | 1p36.31 | 1.9995 | 2.1227 | SPATA7 | 14q31.3 | 1.9974 | 2.1381 |
| INTS8 | 8q22.1 | 2.3634 | 2.167 | TTC8 | 14q31.3 | 2.0015 | 2.1972 |
| BRD7 | 16q12 | 1.9897 | 2.154 | TPD52 | 8q21 | 2.4283 | 2.323 |
| C8orf37 | 8q22.1 | 2.3605 | 2.1766 | ZBTB10 | 8q21.13 | 2.432 | 2.323 |
| PLEKHF2 | 8q22.1 | 2.3634 | 2.1766 | TRAPPC6B | 14q21.1 | 2.2548 | 2.2152 |
| SH3BGR | 21q22.3 | 1.9681 | 2.1224 | TBC1D15 | 12q21.1 | 2.0764 | 2.1976 |
| ZFAT | 8q24.22 | 2.4229 | 2.2267 | RAB21 | 12q21.1 | 2.077 | 2.1976 |
| MLLT10 | 10p12 | 2.0481 | 1.9358 |  |  |  |  |

* genes are sorted according to their contribution to the classification model.

**Table S5. KEGG pathways analysis for GE signatures**

| No. | KEGG ID | Pathways | *p*-value | Genes |
| --- | --- | --- | --- | --- |
| 1 | hsa05219 | Bladder cancer | 0.0057 | EGFR, KRAS, MDM2 |
| 2 | hsa05223 | Non-small cell lung cancer | 0.0093 | EGFR, FHIT, KRAS |
| 3 | hsa05214 | Glioma 3 | 0.0125 | EGFR, KRAS, MDM2 |
| 4 | hsa04144 | Endocytosis | 0.0126 | EGFR, RET, ERBB3, MDM2 |
| 5 | hsa05218 | Melanoma | 0.0157 | EGFR, KRAS, MDM2 |
| 6 | hsa04012 | ErbB signaling pathway | 0.0230 | EGFR, KRAS, ERBB3 |
| 7 | hsa05215 | Prostate cancer | 0.0240 | EGFR, KRAS, MDM2 |

**Table S6. KEGG pathways analysis for ME signatures**

| No. | KEGG ID | Pathways | *p*-value | Genes |
| --- | --- | --- | --- | --- |
| 1 | hsa05218 | Melanoma | 2.529E-4 | EGFR, KRAS, MET, FGF11, MDM2 |
| 2 | hsa05200 | Pathways in cancer | 4.339E-4 | EGFR, RET, KRAS, MET, PAX8, FGF11, MDM2, STAT1 |
| 3 | hsa05214 | Glioma | 0.0027 | EGFR, KRAS, CALML3, MDM2 |
| 4 | hsa05216 | Thyroid cancer | 0.0073 | RET, KRAS, PAX8 |
| 5 | hsa04144 | Endocytosis | 0.0086 | EGFR, RET, ERBB3, MET, MDM2 |
| 6 | hsa05219 | Bladder cancer | 0.0151 | EGFR, KRAS, MDM2 |
| 7 | hsa05223 | Non-small cell lung cancer | 0.0243 | EGFR, FHIT, KRAS |
| 8 | hsa05212 | Pancreatic cancer | 0.0413 | EGFR, KRAS, STAT1 |
| 9 | hsa04020 | Calcium signaling pathway | 0.0434 | EGFR, CALML3, ERBB3, HTR2B |

**Table S7. KEGG pathways analysis for CNV signatures**

| **No.** | **KEGG ID** | **Pathways** | ***p*-value** | **Genes** |
| --- | --- | --- | --- | --- |
| 1 | hsa05200 | Pathways in cancer | 0.0017 | MDM2, MET, RET, STAT1, KRAS |
| 2 | hsa04144 | Endocytosis | 0.0018 | ARFGAP3, MDM2, MET, RET |
| 3 | hsa05218 | Melanoma | 0.0020 | MDM2, MET, KRAS |
| 4 | hsa04120 | Ubiquitin mediated proteolysis | 0.0068 | MDM2, MGRN1, UBR5 |
| 5 | hsa05216 | Thyroid cancer | 0.0088 | RET, KRAS |

**References**

1. Wenzlaff, A.S., M.L. Cote, C.H. Bock, S.J. Land, and A.G. Schwartz, *GSTM1, GSTT1 and GSTP1 polymorphisms, environmental tobacco smoke exposure and risk of lung cancer among never smokers: a population-based study.* Carcinogenesis, 2005. **26**(2): p. 395-401.

2. Torres-Duran, M., A. Ruano-Ravina, K.T. Kelsey, I. Parente-Lamelas, V. Leiro-Fernandez, I. Abdulkader, M. Provencio, J. Abal-Arca, O. Castro-Anon, C. Montero-Martinez, I. Vidal-Garcia, M. Amenedo, A. Golpe-Gomez, C. Martinez, R. Guzman-Taveras, M.J. Mejuto-Marti, A. Fernandez-Villar, and J.M. Barros-Dios, *Environmental tobacco smoke exposure and EGFR and ALK alterations in never smokers' lung cancer. Results from the LCRINS study.* Cancer Lett, 2017. **411**: p. 130-135.

3. Wu, J.Y., J. Wang, J.C. Lai, Y.W. Cheng, K.T. Yeh, T.C. Wu, C.Y. Chen, and H. Lee, *Association of O6-methylguanine-DNA methyltransferase (MGMT) promoter methylation with p53 mutation occurrence in non-small cell lung cancer with different histology, gender, and smoking status.* Ann Surg Oncol, 2008. **15**(11): p. 3272-7.

4. Blumenschein, G.R., Jr., E.F. Smit, D. Planchard, D.W. Kim, J. Cadranel, T. De Pas, F. Dunphy, K. Udud, M.J. Ahn, N.H. Hanna, J.H. Kim, J. Mazieres, S.W. Kim, P. Baas, E. Rappold, S. Redhu, A. Puski, F.S. Wu, and P.A. Janne, *A randomized phase II study of the MEK1/MEK2 inhibitor trametinib (GSK1120212) compared with docetaxel in KRAS-mutant advanced non-small-cell lung cancer (NSCLC)dagger.* Ann Oncol, 2015. **26**(5): p. 894-901.

5. Krystal, G.W., C.S. DeBerry, D. Linnekin, and J. Litz, *Lck associates with and is activated by Kit in a small cell lung cancer cell line: inhibition of SCF-mediated growth by the Src family kinase inhibitor PP1.* Cancer Res, 1998. **58**(20): p. 4660-6.

6. Camidge, D.R. and K.D. Davies, *MET Copy Number as a Secondary Driver of Epidermal Growth Factor Receptor Tyrosine Kinase Inhibitor Resistance in EGFR-Mutant Non-Small-Cell Lung Cancer.* J Clin Oncol, 2019. **37**(11): p. 855-857.

7. Song, K., J.-H. Bi, Z.-W. Qiu, R. Felizardo, L. Girard, J.D. Minna, and A.F. Gazdar, *A quantitative method for assessing smoke associated molecular damage in lung cancers.* Translational Lung Cancer Research, 2018. **7**(4): p. 439-449.

8. Wang, Y., X. Peng, L. Zhu, L. Hu, and Y. Song, *Genetic variants of CHRNA5-A3 and CHRNB3-A6 predict survival of patients with advanced non-small cell lung cancer.* Oncotarget, 2016. **7**(18): p. 26436-43.

9. Kang, M.W., E.S. Lee, S.Y. Yoon, J. Jo, J. Lee, H.K. Kim, Y.S. Choi, K. Kim, Y.M. Shim, J. Kim, and H. Kim, *AKR1B10 is associated with smoking and smoking-related non-small-cell lung cancer.* J Int Med Res, 2011. **39**(1): p. 78-85.

10. Ghosh, A. and H. Yan, *Hydrogen bond analysis of the EGFR-ErbB3 heterodimer related to non-small cell lung cancer and drug resistance.* J Theor Biol, 2019. **464**: p. 63-71.

11. Li, W. and F. He, *Monocyte to macrophage differentiation-associated (MMD) targeted by miR-140-5p regulates tumor growth in non-small cell lung cancer.* Biochem Biophys Res Commun, 2014. **450**(1): p. 844-50.

12. Yang, L., Y. Dong, Y. Li, D. Wang, S. Liu, D. Wang, Q. Gao, S. Ji, X. Chen, Q. Lei, W. Jiang, L. Wang, B. Zhang, J.J. Yu, and Y. Zhang, *IL-10 derived from M2 macrophage promotes cancer stemness via JAK1/STAT1/NF-kappaB/Notch1 pathway in non-small cell lung cancer.* Int J Cancer, 2019.

13. Dudnik, E., J. Bar, N. Peled, E. Bshara, T. Kuznetsov, A.Y. Cohen, T. Shochat, H. Nechushtan, A. Onn, A. Agbarya, M. Moskovitz, S. Keren, N. Popovits-Hadar, D. Urban, M. Mishaeli, N.M. Rabinovich, R. Brenner, A. Zer, O. Rotem, L.C. Roisman, M. Wollner, and G. Israel Lung Cancer, *Efficacy and Safety of BRAF Inhibitors With or Without MEK Inhibitors in BRAF-Mutant Advanced Non-Small-Cell Lung Cancer: Findings From a Real-Life Cohort.* Clin Lung Cancer, 2019.

14. Javid, J., R. Mir, P.K. Julka, P.C. Ray, and A. Saxena, *Association of p53 and mdm2 in the development and progression of non-small cell lung cancer.* Tumour Biol, 2015. **36**(7): p. 5425-32.

15. Wang, S., J. Wang, Y. Bai, Q. Wang, L. Liu, K. Zhang, X. Hong, Q. Deng, X. Zhang, M. He, T. Wu, P. Xu, and H. Guo, *The genetic variations in DNA repair genes ERCC2 and XRCC1 were associated with the overall survival of advanced non-small-cell lung cancer patients.* Cancer Med, 2016. **5**(9): p. 2332-42.

16. Geng, X., W. Pu, Y. Tan, Z. Lu, A. Wang, L. Tan, S. Chen, S. Guo, J. Wang, and X. Chen, *Quantitative assessment of the diagnostic role of FHIT promoter methylation in non-small cell lung cancer.* Oncotarget, 2017. **8**(4): p. 6845-6856.

17. Wikman, H., S. Thiel, B. Jager, P. Schmezer, B. Spiegelhalder, L. Edler, H. Dienemann, K. Kayser, V. Schulz, P. Drings, H. Bartsch, and A. Risch, *Relevance of N-acetyltransferase 1 and 2 (NAT1, NAT2) genetic polymorphisms in non-small cell lung cancer susceptibility.* Pharmacogenetics, 2001. **11**(2): p. 157-68.

18. Moncho-Amor, V., L. Pintado-Berninches, I. Ibanez de Caceres, E. Martin-Villar, M. Quintanilla, P. Chakravarty, M. Cortes-Sempere, B. Fernandez-Varas, C. Rodriguez-Antolin, J. de Castro, L. Sastre, and R. Perona, *Role of Dusp6 Phosphatase as a Tumor Suppressor in Non-Small Cell Lung Cancer.* Int J Mol Sci, 2019. **20**(8).

19. Furugaki, K., M. Mochizuki, M. Kohno, S. Shu, N. Harada, and Y. Yoshimura, *Expression of C-terminal ALK, RET, or ROS1 in lung cancer cells with or without fusion.* BMC Cancer, 2019. **19**(1): p. 301.

20. Gao, X.Z., G.N. Wang, W.G. Zhao, J. Han, C.Y. Diao, X.H. Wang, S.L. Li, and W.C. Li, *Blocking OLFM4/HIF-1alpha axis alleviates hypoxia-induced invasion, epithelial-mesenchymal transition, and chemotherapy resistance in non-small-cell lung cancer.* J Cell Physiol, 2019.

21. Cooper, W.N., R.E. Dickinson, A. Dallol, E.V. Grigorieva, T.V. Pavlova, L.B. Hesson, I. Bieche, M. Broggini, E.R. Maher, E.R. Zabarovsky, G.J. Clark, and F. Latif, *Epigenetic regulation of the ras effector/tumour suppressor RASSF2 in breast and lung cancer.* Oncogene, 2008. **27**(12): p. 1805-11.
